# Left ventricular hypertrophy amplifies the QT, and Tp-e intervals and the Tp-e/ QT ratio of left chest ECG

**DOI:** 10.1016/S1674-8301(10)60011-5

**Published:** 2010-01

**Authors:** Zhao Zhao, Zuyi Yuan, Yuqiang Ji, Yue Wu, Yinzhi Qi

**Affiliations:** Department of Cardiology, Key Laboratory of Environmental Genes Related to Diseases, Ministry of Education, the First Affiliated Hospital of Medical College, Xi'an Jiaotong University, Xi'an 710061, China.

**Keywords:** hypertension, left ventricular hypertrophy, QT interval, Tp-e interval, arrhythmia

## Abstract

**Objective:**

To evaluate the changes in Tp-e interval (an interval from the peak to the end of the T wave), QT interval and Tp-e/QT ratio of the body surface ECG in patients with left ventricular hypertrophy (LVH).

**Methods:**

The Tp-e interval and QT interval were measured on body surface ECGs in 42 patients without either hypertension or LVH (control group), 41 patients having hypertension but not LVH (non-LVH group), and 38 patients with both hypertension and LVH (LVH group).

**Results:**

The mean corrected QT (QTc) interval, and mean corrected Tp-e[T(p-e)c] interval were significantly longer in the LVH group (0.430±0.021s vs. 0.409±0.019s, *p* < 0.01; 0.098±0.013s vs. 0.088±0.011s, respectively) than those in the control group. The Tp-e/QT ratio was also amplified in LVH group (0.232±0.028 vs.0.218±0.027) (*p* < 0.05).

**Conclusion:**

LVH increased the QT interval, Tp-e interval and Tp-e/QT ratio of the body surface ECG.

## INTRODUTION

Left ventricular hypertrophy (LVH) is a well-recognized risk factor for cardiovascular disease, and is associated with malignant ventricular arrhythmias, leading to sudden cardiac death (SCD)[1],[2]. Several animal studies have demonstrated that LVH can lead to alterations of ion channel density and expression, prolonged action potential duration and transmural dispersion of repolarization (TDR)[3],[4].

Amplification of TDR has long been known as a substrate for ventricular arrhythmias[5]. The Tp-e interval correlates well with TDR, whereas the QT interval is closely approximated by the midmyocardial cell (M cell) action potential duration[6]. Thus the purpose of this study is to investigate the changes in the QT and Tp-e intervals and Tp-e/QT ratio in the left chest leads of the body surface ECG in patients with LVH.

## MATERIALS AND METHODS:

### Clinical materials

One hundred and twenty-one inpatients from March 2008 to September 2008 in the First Affiliated Hospital of Medical College of Xi'an Jiaotong University were enrolled into the study. All patients underwent echocardiography to determine LVH. Hypertension was diagnosed according to the JNC7 criteria[7], and LVH was defined by any of the following three ECG criteria[8]: ① LVMI > 125 g/m^2^ (male), > 110 g/m^2^ (female); ② LVPWT ≥ 12 mm; ③ IVST ≥ 12 mm, where LVMI, LVPWT and IVST denote left ventricular mass index[9], left ventricular posterior wall thickness, and interventricular septal thickness, respectively. According to the criteria of hypertension and LVH, patients were divided into 3 groups: ① control group (*n* = 42), patients without LVH and with no history of hypertension; ② non-LVH group (*n* = 41), patients without LVH but with a history of hypertension, ③ LVH group (*n* = 38), patients with LVH and a history of hypertension **(*Table 1*)**.

None of the study subjects were treated with agents that could affect the QT interval. Patients with secondary hypertension, electrolyte disorders, atrial fibrillation, bundle branch block, atrioventricular block, Wolff-Parkinson-White syndrome, myocardial infarction, or who had pacemakers were excluded from the study.

**Table 1:** Clinical characteristics of patients in the three groups

	Control group	non-LVH group	LVH group
Number of patients	42	41	38
Age (years)	57.70 ± 11.40	56.90 ± 12.70	58.30 ± 14.20
Number of male	23	23	20
Serum potassium (mEq/L)	4.24 ± 0.22	4.20 ± 0.31	4.22 ± 0.38
Heart rate (time/min)	68.20 ± 10.40	66.40 ± 14.80	65.90 ± 12.30

There were no significant differences between groups.

### Electrocardiographic measurements

A standard 12-lead ECG was recorded at 25 mm/s, 1 mV/cm calibration. All subjects were in sinus rhythm. Electrocardiographic intervals were measured manually by an experienced physician using the lead V_4_-V_6_. The QT interval was defined as the time from the onset of the QRS complex to the end of the T wave at which the isoelectric line intersected a tangential line drawn at the maximal down slope of a positive T wave. The QTpeak interval was measured as the time from the onset of QRS complex to the point at the peak of a positive T wave. The difference between the QT and QTpeak intervals was taken as the Tp-e interval. Three consecutive QT and QTpeak intervals were measured and averaged. All the intervals were corrected for heart rate by using Bazett's formula[10]. The low-amplitude T wave (amplitude < 0.1 mV), inverted T wave, biphasic and bifurcated T wave (interval between two peaks > 0.15 s) were excluded.

### Statistical analysis

Data are presented as mean±SD. Data analyses were performed by using SPSS11.5 software. One-way analysis of variance was used to determine statistical significance of the differences in QT, QTpeak, Tp-e interval and Tp-e/QT ratio among the three groups. A value of *P* < 0.05 was considered statistically significant.

## RESULTS

### Patient characteristics

There were no significant differences in age, gender, serum potassium concentration or heart rate among the three groups **(*Table 1*)**.

### Comparison of the corrected QT peak, QT and T(p-e)c interval among the three groups

**Fig. 1:**
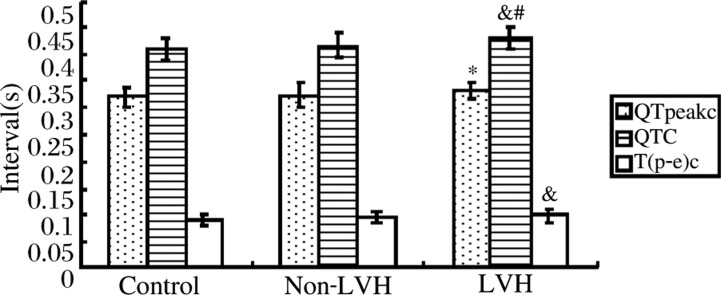
The QTpeakc, QTc and T(p-e)c intervals compared among the three groups. Compared with control group, *P < 0.05, ^&^*P* < 0.01; comparison of non-LVH group with LVH group, ^#^*P* < 0.05.

The mean corrected QTpeak (QTpeakc) interval, and QT(QTc) interval were significantly longer in the LVH group than these in the control group, especially in the case of the QTc interval. Compared with the control group, the non-LVH group exhibited no QTpeakc interval or QTc interval prolongation. When comparing the two groups with hypertension, there was a significant increase in the QTc interval in LVH group **(*Table 2**,**Fig. 1*)**.

The mean corrected Tp-e [T(p-e)c] interval was significantly increased in the LVH group when compared to the control group, because of preferential prolongation of the QT (QTc) interval. There was no statistically significant difference in T(p-e)c interval between the control group and non-LVH group, or between the non-LVH group and LVH group (*p* = 0.052) ***(Table 2, Fig. 1)***.

### Comparison of the T(p-e)c/QTc ratio among the three groups

**Fig. 2:**
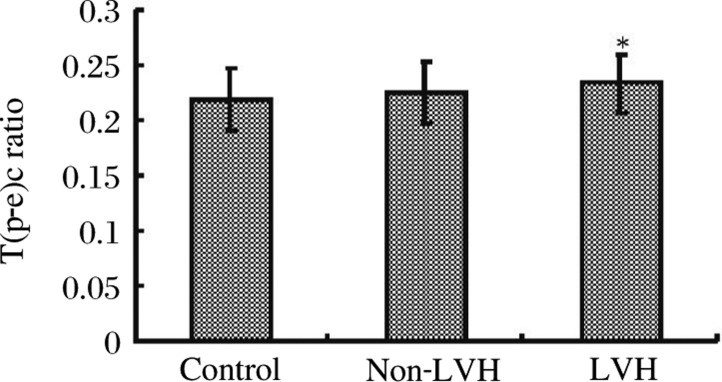
The T(p-e)c/QTc ratio compared among the three groups. Compared with control group, *P < 0.05.

The mean T(p-e)c/QTc ratios in the control group, non-LVH group and LVH group were 0.218±0.027, 0.225±0.028, and 0.232±0.028, respectively. This upward trend in the T(p-e)c/QTc ratio was only statistically significant when the LVH group value was compared with the control group (*p* < 0.05). There was no significant difference between the non-LVH and LVH groups (***Table 2******,*** ***Fig. 2***).

**Table 2:** The QTpeakc, QTc and T(p-e)c intervals of the three patient groups

	Control group	Non-LVH group	LVH group
QTpeakc interval(s)	0.321 ± 0.019	0.324 ± 0.023	0.332 ± 0.015*
QTc interval(s)	0.409 ± 0.019	0.417 ± 0.023	0.430 ± 0.021^&#^
T(p-e)c interval(s)	0.088 ± 0.011	0.093 ± 0.010	0.098 ± 0.013^&^
T(p-e)c/QTc ratio	0.218 ± 0.027	0.225 ± 0.028	0.232 ± 0.028*

Compared with control group, *P < 0.05, ^&^*P* < 0.01; comparison of non-LVH group with LVH group, ^#^*P* < 0.05.

## DISCUSSION

The chief findings of the present study are that ① the LVH group had longer QTpeak, QT and Tp-e intervals than the control group, which reflect the repolarization in the left ventricles, and ② the Tp-e/QT ratio is amplified in the LVH group.

A few studies have demonstrated that LVH resulted in a marked increase in ventricular repolarization time[11],[12]. Because the end of repolarization of the M cells coincides with the end of the T wave, and the end of repolarization of the epicardial cells coincides with the peak of the T wave, it is not surprising that the QTpeak and QT intervals in the LVH group are longer than those in the control group. Although the underlying mechanism is unknown, it is probably associated with the increased wall thickness. This is further supported by pulmonary hypertensive patients who have longer QTpeak and QT intervals in the right chest leads of the body surface ECG[13].

Another important finding of this study is that Tp-e and Tp-e/QT ratio is amplified in the LVH group. This is likely because of the disproportional prolongation of the Tp-e interval relative to QT interval in LVH patients. This indicates that the Tp-e interval, like the QT interval, is also positively correlated to ventricular wall thickness.

The Tp-e interval has been accepted as an easy, assessable measure of TDR, which is related to arrhythmogenesis. This suggests that in LVH there is a high transmural heterogeneity of repolarization, and hence has high arrhythmic risk.

We provide the first evidence that LVH leads to a notable increase in the Tp-e/QT ratio. Several studies have demonstrated that the ratio may serve as a good clinical predictor of SCD in long QT (LQT) syndrome and other ion channel diseases [14],[15]. As LVH is similar to acquired LQT syndrome in some aspects[16], it is easy to understand the high incidence of SCD in LVH patients.

The Tp-e and QT intervals vary greatly with heart rate and across species. However, the Tp-e/QT ratio remains relatively constant in a narrow range of values[14]. Drugs blocking I_Kr_ or enhancing late I_Na_ or I_Ca-L_ cause an increase in the Tp-e/QT ratio, and vice versa[17]. By using the arterially perfused rabbit ventricular wedge preparation, Liu *et al*[18] found that azithromycin significantly prolonged both the QT and Tp-e intervals without changing the Tp-e/QT ratio, and at higher doses carried a much smaller torsade de pointes (TdP) risk than those agents amplifying the Tp-e/QT ratio. More interestingly, Yamaguchi *et al*[19]demonstrated that a Tp-e/QT ratio exceeding 0.28 was tightly correlated with the risk of developing TdP in patients with acquired LQT syndrome.

Some researchers also observed the effect of LVH on QT and T(p-e) intervals in left chest leads[20]. However, they did not investigate the change in Tp-e/QT ratio or measure the heart rate and serum potassium among the patients. Because age, gender and electrolyte disturbance[21],[22] have obvious effects on the repolarization time, they were matched in our three groups.
